# Lessons learned on social health integration: evaluating a novel social health integration and social risk-informed care online continuing professional development course for primary care providers

**DOI:** 10.1186/s12909-025-06971-9

**Published:** 2025-04-08

**Authors:** Elizabeth Bojkov, Dea Papajorgji-Taylor, Andrea R. Paolino, Caitlin N. Dorsey, Katheen A. Barnes, Meagan C. Brown

**Affiliations:** 1 University of Washington School of Public Health, Seattle, WA USA; 2 Kaiser Permanente Northwest Center for Health Research, Portland, OR USA; 3 Kaiser Permanente Colorado Institute for Health Research, Aurora, CO USA; 4 Kaiser Permanente Washington Health Research Institute, 1730 Minor Ave, STE 1600, Seattle, WA 98101 USA; 5 Washington Permanente Medical Group, Seattle, WA USA

**Keywords:** Social needs, Social risks, Social health, Standard of care, Social determinants of health, Training, Quality of healthcare, Evaluation, CPD, Qualitative

## Abstract

**Background:**

Adjusting clinical care to account for social risks and needs is vital to patient-centered care, but little attention has been paid to implementing it in routine practice. Kaiser Permanente co-designed and developed a continuing professional development (CPD) course to orient providers to adjustment activities, or *social risk-informed care.* We evaluated the dissemination and implementation of this course.

**Methods:**

We evaluated the dissemination and implementation of the online CPD using the RE-AIM implementation framework and the Kirkpatrick model of evaluation for training and learning programs. Administrative records and completion reports were generated to track dissemination and completion. A pre- and post-survey design was utilized to assess provider changes in knowledge, attitudes, beliefs, and self-efficacy in delivering social risk-informed care, and semi-structured interviews were conducted to describe effectiveness of the online CPD, adoption of social risk-informed care, and sustainability of the online CPD and other Kaiser Permanente social health integration initiatives.

**Results:**

From April 2022-February 2023, 82 individuals completed the online CPD; 52 participants completed the pre-survey and 38 completed the post-survey. A total of 17 interviews were conducted over two phases of qualitative data collection (passive dissemination versus active dissemination). Interviewees felt the online CPD provided foundational knowledge in social health and social risk-informed care but requested more region- and role-specific resources. They also identified several systems-level barriers to social health integration.

**Conclusions:**

Co-designing medical education courses with various stakeholders is vital to ensuring relevant and effective educational material. However, high-quality, intentionally designed educational material needs to be complemented with multifaceted and targeted implementation strategies to achieve intended provider behavior change and improved patient outcomes.

**Supplementary Information:**

The online version contains supplementary material available at 10.1186/s12909-025-06971-9.

## Background

Social factors account for a majority of overall health status and disparities in health [1–4]. Prior studies have demonstrated that social factors contribute to between 40%−70% of individuals' health status; in contrast, healthcare accounts for approximately 20% [8–11]. Social factors both directly and indirectly impact health and health disparities. For example, transportation challenges cause missed appointments [5, 6], while housing instability increases medication non-adherence and avoidable healthcare utilization among low-income populations [7–10]. Other social risks such as food insecurity directly impact the incidence, progression, and outcomes of chronic diseases such as diabetes and cancer [11, 12]. As a result, national organizations like the National Committee for Quality Assurance (NCQA) and Centers for Medicare and Medicaid Services (CMS) are implementing standards and quality metrics for healthcare organizations to address social risk factors through screening and referral to resources [13, 14]. The National Academy of Medicine (NAM) identified five priorities for integrating care for social factors into healthcare systems, or social health integration: awareness, adjustment, assistance, alignment, and advocacy [15]. Kaiser Permanente has used the report as a guide to develop and implement social health initiatives to address the root causes of health disparities.

Kaiser Permanente is an integrated healthcare system that serves 12.7 million members in eight regional markets (California, Colorado, Georgia, Hawaii, Maryland, Virginia, Oregon, Washington DC, Washington State). Over the past several years, Kaiser Permanente has increased support for social health screening *(awareness)* and social services referral workflows and systems *(assistance) *[16]. However, little attention has been given to how clinicians can adapt care to account for patients’ social risks, an approach known as social risk-informed care [15]. Social risk-informed care, or adjusting care to account for social risk factors, has been shown to improve patient-centeredness of care and reduce provider burnout [17–20]. The nascent body of literature examining the impact of specific social risk-informed care strategies have also demonstrated improvements in patient health outcomes [21]. Strategies associated with improved patient outcomes such as improved medication adherence and fewer missed appointments and emergency department visits include switching to mail-order prescriptions and telehealth visits for patients with transportation challenges, opting for generic medication alternatives for patients experiencing financial strain, titrating insulin for patients with food insecurity, and opting for medications that do not need refrigeration for patients with unstable housing [17, 21, 22].

In 2021, Kaiser Permanente’s Social Needs Network for Evaluation and Translation (SONNET) released a foundational report on the state of the evidence for social risk-informed care [23]. The report generated several recommendations for future practice, including the development of provider trainings to support uptake of social risk-informed care [23]. In response, the online, asynchronous training entitled, ‘Addressing Social Health in Medicine,’ was co-developed with researchers, providers, and patients. The objective of this study was to evaluate the implementation and effectiveness of the online continuing professional development (CPD) using the RE-AIM framework [24].

## Methods

### CPD development

The Addressing Social Health in Medicine online CPD was co-designed from July 2021-April 2022 and engaged four groups: a team of clinicians and researchers experienced in professional development and design of online CPDs; an advisory committee that provided subject matter expertise and feedback on training content and case study design from within and outside of Kaiser Permanente; members of the target audience (primary care providers) that provided feedback on a one-on-one basis; and a patient panel that was demographically representative (race, ethnicity, gender, age and parental status) that met to provide feedback on Kaiser Permanente’s approach to engaging with social health data and care adjustment [25]. Given the lack of evidence-based guidelines for specific adjustments, our team employed a user-centered framework called Discover, Design/Build, and Test to iteratively develop the online CPD. This approach allowed the core design team to identify patient and provider priorities and perspectives on how to present social health integration and care adjustment in the training given the current state of the field [26]. Prior to launch, the final product was tested with end users (primary care physicians, a nurse practitioner, and a patient navigator). Details regarding the co-design process have been published previously [13].

The CPD was targeted towards clinicians with the aim of building the skills needed to practice social risk-informed care with patients in their clinical practice. These skills include: recognizing verbal and nonverbal cues that suggest a patient is experiencing a social risk, and initiating sometimes sensitive conversations in order to collaborate with the patient about care adjustment that supports the patients' health goals [17]. Based on feedback from the four co-design groups, the online CPD included three components: 1) an introduction to the importance of social health and contextualized care, including a four-step framework to help providers identify and address a patient’s social risks; 2) an overview of organizational social health integration strategy; and 3) three interactive and skill-focused social risk-informed care case studies. Part 1 introduces why social health matters and provides a four-step framework based on prior studies [17, 18], including listening for clues that indicate a patient has a social risk that may be impacting care, asking questions to find out more, reflecting back to the patient to make sure you understand the social risks and how they are impacting the patient’s ability to follow through on care plans, and working with the patient to adapt their care plan based on risks. Unlike approaches that focus solely on screening or referral, this framework emphasizes adjusting clinical care plans to accommodate patients’ social circumstances.

Part 2 of the course offers an overview of organizational strategies for social health integration, ensuring that clinicians understand both the larger system-level context and region-specific Kaiser Permanente resources. Part 3 presents two interactive case studies, co-developed with practicing clinicians from the advisory committee based on real-world examples, that allow learners to apply the four-step process in scenarios reflecting the complexity of real-world social risk-informed care. One case study focuses on a patient with diabetes who is experiencing food insecurity and financial strain, impacting her ability to afford glucose testing strips. For this patient, the recommended adjustments are to consider titrating the insulin dosage and timing as well as glucose testing frequency and timing. The second case study focused on an adolescent who has been having trouble with adhering to their asthma medication due to her parents’ separation and living between two houses. In this situation, the provider obtains authorization for a second controller and rescue inhaler to enable the adolescent to have their medications at both locations. Throughout the course, patient perspectives and evidence support the notion that these adjustments can ultimately save time and improve care [25].

To reinforce key lessons, our team also worked with providers on the advisory committee to develop supplementary “tip sheets” and provided opportunities for clinicians to identify local resources that support social health integration in their regions [25]. Altogether, this CPD training moves beyond awareness and assistance into an opportunity for clinicians learn how to operationalize social risk adjustment strategies in everyday practice [25].

### CPD evaluation

Our team utilized the RE-AIM framework to determine the research questions for the evaluation (Table 1) [24, 27]. The RE-AIM framework helps evaluators understand the real-world impact of interventions through examining reach (the number or proportion of individuals who engaged in the intervention), effectiveness (the real-world impact of implementing the intervention), adoption (the organizational or settings-level factors that contribute to changes in behavior, policy or practice), implementation (whether the intervention was implemented as intended), and maintenance (the sustainability of the intervention’s impact) [27]. To further define effectiveness in the context of the asynchronous online CPD, the Kirkpatrick model for evaluating employee training was also used to inform the evaluation (Table 2) [28]. In particular, Level 2 (Learning) of the Kirkpatrick model focuses on the changes in knowledge, attitudes and belief, and confidence in new skills acquired from the targeted training, and Level 3 (Behavior) seeks to understand whether participants applied new knowledge and skills obtained through the training [28]. This evaluation was deemed a quality improvement project and exempt by regional Kaiser Permanente Institutional Review Boards prior to beginning this work.

**Table 1:** RE-AIM for the evaluation of ‘Addressing Social Health in Medicine’ online CPD

RE-AIM Domain	Meaning	Evaluation Question	Data Source
Reach	The absolute number, proportion or representativeness of the population that participates in an intervention or initiative	1. How many providers took the CPD? 2. What was the distribution by role and region? 3. How did providers hear about the CPD? 4. What proportion of those reached through dissemination completed the CPD?	completion reports, training-embedded survey
Effectiveness	The impact of the intervention on important outcomes, including negative consequences, quality of life and economic outcomes	1. Did the CPD lead to changes in providers’ Knowledge, Attitudes, Beliefs, Confidence and Self-Efficacy for SRIC? (Kirkpatrick Levels 2 and 3) 2. Did the CPD lead to new or improved application of SRIC skills in providers’ routine practice?	training-embedded survey, provider interviews
Adoption	The absolute number, proportion or representativeness of settings or individuals willing to initiate an intervention	1. What are a) organizational and b) individual level cultural and climate factors that facilitate translation of training into changed provider behavior and implementing social health?	provider interviews
Implementation	At the settings level, can be thought of as fidelity to the way the intervention was intended to be implemented	1. How many participants: completed the CPD, completed free-text responses, completed the surveys, and completed trackable clicks during the CPD? 2. Online CPD completion time	completion reports, training-embedded survey
Maintenance	To what extent is an intervention institutionalized or folded into routine practice	1. To what extent has provider behavior change been sustained?	training-embedded survey, provider interviews

**Table 2:** Kirkpatrick model levels 1–4

Level	Description
Level 1: Reaction	Do participants find the training “favorable, engaging and relevant to their jobs?” [17]
Level 2: Learning	Did participants acquire the, “the intended knowledge, skills, attitude, confidence, and commitment based on their participation in the training?” [17]
Level 3: Behavior	Did participants “apply what they learned during training when they are back on the job?” [17]
Level 4: Results	Did the desired results occur? If so, how well?

### Quantitative data collection and methods

The training was disseminated to Kaiser Permanente staff through email, social health workgroup presentations, and newsletters. We assessed dissemination using counts of members in these various audiences. Newsletter recipients represented a broad range of individuals across Kaiser Permanente interested social health, including researchers, providers, and staff. Email and presentation outreach was more targeted and focused on outreach to physicians and other healthcare providers across the organization. For newsletter and email outreach, subscriber lists and email addresses were used to estimate the audience size. For presentations, dissemination was assessed by the number of individuals in attendance. We were unable to assess the degree of overlap between newsletter and other dissemination activities; as a result audience sizes may not represent unique counts.

To assess the number of participants who completed the training and their role, completion reports were automatically generated weekly by the learning platform. The completion reports included name, job role, region, employee type (employee vs physician), CPD completion status and date of completion. Finally, we utilized a Qualtrics survey with several close-ended and open-ended questions for participants to complete immediately before and immediately after the CPD. The pre-survey included two demographic questions (region and role) and 4 Likert-scale questions assessing participants’ knowledge, attitude and beliefs of participants before and after completing the CPD. The post-survey also included one 10-point scale question asking how likely the participant would be to recommend the CPD, one multiple-choice question asking how the participant heard about the CPD, and two free-response questions with space to add additional resources and topics they wished the CPD covered more in depth.

### Qualitative data collection and methods

To evaluate the online CPD’s effectiveness and adoption qualitatively, interviews were conducted with participants who completed the CPD between April 8th and October 31st, 2022.

The interview guide was developed based on RE-AIM domains of effectiveness, adoption, and maintenance, as well as the Kirkpatrick Model framework of training evaluation Levels 2 and 3 (Learning and Behavior). Drafts were reviewed with the evaluation team with experience in social risk-informed care and piloted with a Kaiser Permanente clinician involved in the development of social health initiatives and the co-design and development of the online CPD [25]. Prior to finalizing the guide, evaluators sought input from partners at the regional and national levels. The final interview guide can be found in Appendix 1.

### Interview participant recruitment

Qualitative interviews occurred over two phases.

#### Phase 1

Semi-structured interviews with participants who completed the online CPD between April-August 2022 were conducted to assess participant perceptions of effectiveness, adoption of social risk-informed care concepts into clinical practice and their perspectives on the sustainability of the CPD and social health integration. Eligible participants were identified via Kaiser Permanente’s internal education platform and outreach was conducted via email invitation. Interviews were approximately 30–45 min long, audio recorded and transcribed verbatim.

#### Phase 2

To increase the diversity of perspectives based on experience with social risk-informed care, the evaluation team also pursued a more targeted approach by reaching out to physicians who were previously interviewed as part of a 2021 SONNET foundational report on social risk-informed care [23]. Nine physicians were asked to complete the training and were subsequently interviewed using the same interview guide as Phase 1 participants. Interviews occurred from September–October 2022, and all audio recordings were transcribed for review and data analysis. The semi-structured interview guide was slightly modified to account for the participants’ previous engagement in the 2021 social risk-informed care project, as well as their familiarity with current local and national social needs initiatives.

### Qualitative data analysis

For Phase 1 analysis, EB used the interview guide to generate initial codes in a deductive fashion. Deductive codes were then applied to each transcript along with inductive codes that were identified as emergent themes during the coding process. To ensure validity in coding methods, one participant’s transcript was coded by both MCB and EB who then met to discuss, compare, and develop consensus on the code definitions. EB then coded the remaining transcripts. After coding, EB developed a qualitative data matrix and populated themes from each transcript to identify overlap within and across transcripts [29]. Based on the matrix, EB then developed memos while reading through the transcripts a final time to identify themes and representative quotes based on evaluation objectives and research questions. Atlas.ti software was utilized for coding and analyses [30].

For Phase 2, Due to the small sample size, a preliminary review and open coding was applied by DPT, who then reviewed the data a second time and applied the relevant codes from the codebook developed for Phase I participants and mirroring the process used in Phase 1. The full study team agreed upon the final set of codes and representative quotes from both phases. The study codebook can be found in Appendix 2.

## Results

### Quantitative results

Quantitative results were drawn from three sources: 1) dissemination and CPD completion reports, and 2). CPD pre and post survey results.

#### Dissemination and CPD completion reports

Dissemination activities began in January 2022, reaching over 2,148 Kaiser Permanente professionals by September 2022. We were unable to measure the size of the target audience for some activities, such as internal websites. Table 3 describes dissemination activities:

**Table 3:** Dissemination efforts to Kaiser permanente professionals

Type of Outreach	Number of Outreach Activities	Audience Size
Newsletter	6	1,423
Email Communications	4	575
Presentations	3	150

From April to February 2023, 172 individuals began the training module and 82 completed the online CPD. 52 participants (63%) completed the pre-survey, and 40 participants (49%) completed the post-survey.

Of the individuals who completed the CPD, 13 (16%) were classified as physicians in their region, 1 (1%) was a contractor and 68 were classified as employees (83%). The employee classification includes roles such as medical resident, nurse practitioner (NP), physician assistant (PA), nurse (RN), social worker (SW). Table 4 describes participants by health profession.

**Table 4:** CPD Participants and CPD survey participants by profession

Profession	Count	Percent	PRE (# of Responses)	PRE (%)	POST (# of Responses)	POST (%)
**Participants by Health Profession**						
Physicians (MD/DO)	13	16%				
Employee (Medical Residents, NP, PA, Pharm, RN, SW, etc.)	68	83%				
Contractor	1	1%				
**Total**	**82**	**100%**				
**Survey Respondents by Profession**						
Physicians (MD/DO)			22	42%	13	33%
Nurse Practitioners (NP)			1	2%	1	3%
Physician Assistant (PA)			3	6%	1	3%
Other			26	50%	25	62%
**Total**			**52**	**100%**	**40**	**100%**

#### Survey results

A total of 76 participants responded to the pre-survey and 43 participants responded to the post-survey. Providers represented a total of 42% (*n* = 22) in the pre-survey and 30% (*n* = 13) in the post-survey. Table 4 describes survey respondents by health profession.

**Fig. 1:**
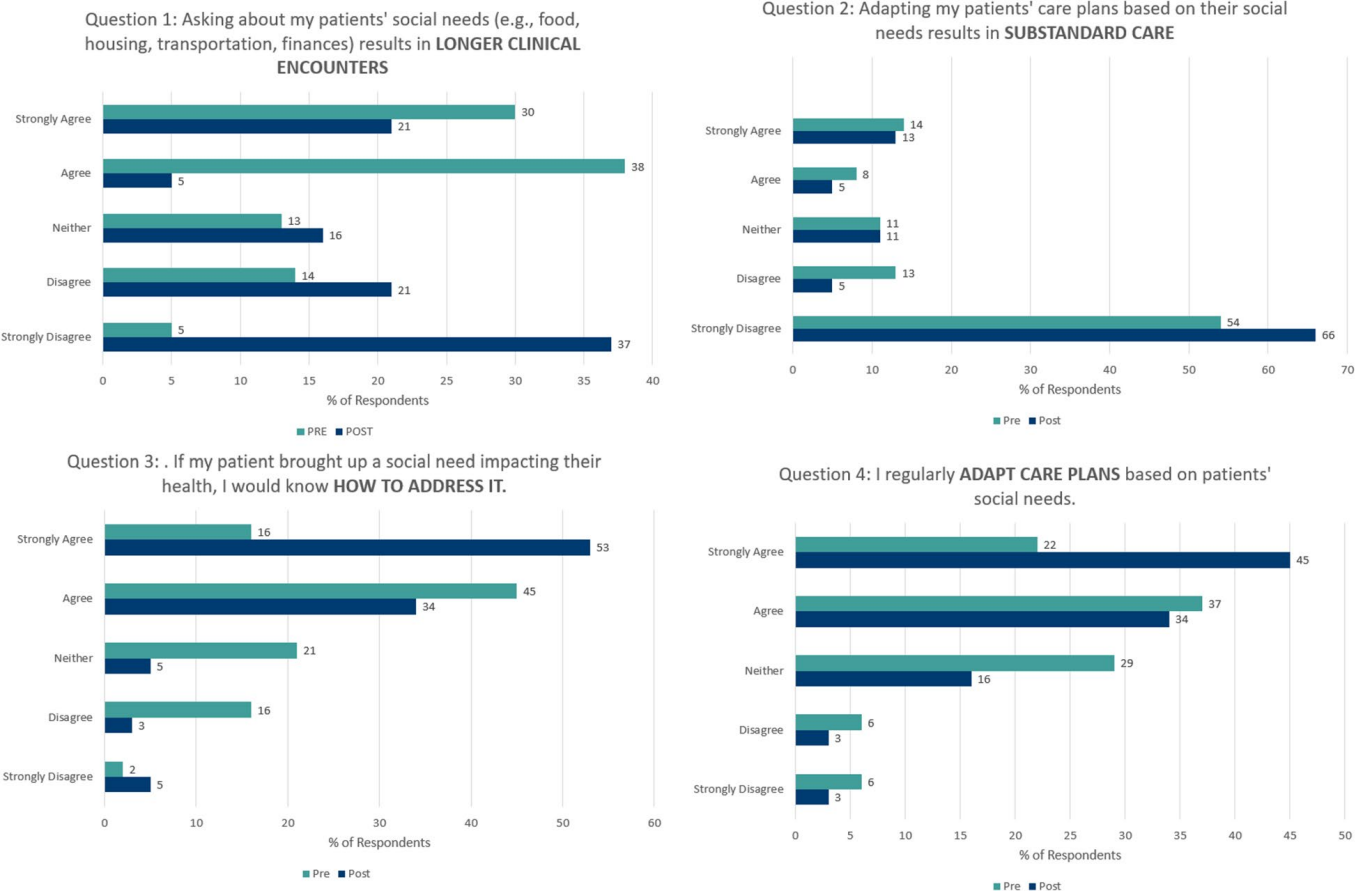
Pre-Post survey results of participants’ knowledge, attitudes, beliefs, and self-efficacy around social risk-informed care

To assess changes in the participants’ knowledge, attitudes, beliefs, and self-efficacy around social risk-informed care, four Likert-style questions were asked both as part of the pre- and post-survey. Results are presented in Fig. 1.

#### Qualitative results

A total of 68 participants were invited to complete interviews in both Phase I and Phase II. Ultimately 17 participants agreed to interviews, for a response rate of approximately 25%. Participant roles can be found in Table 5.

**Table 5:** Interview participants by role

Physicians (MD/DO)	12 (70.6%)
Nurse	1 (5.9%)
Pharmacists	1 (5.9%)
Social Worker	1 (5.9%)
Misc	2 (11.7%)
**Total**	17 (100%)

The primary themes identified during analysis were 1) Word-of-mouth and personal interest drive training engagement; 2) Introduction to new terminology and concepts, case studies based on real-world examples, and role reinforcement are key benefits of the CPD course; 3) A one-size-fits-all approach to training compromises nuance and depth; and 4) Sustainability of learnings requires an ongoing and systems-level approach.

#### Word-of-mouth and personal interest drive training engagement

Participants were asked how they learned about the CPD and what motivated them to complete it. Most heard about the online CPD from a colleague or supervisor. In some cases, participants were required to take it by their supervisors. Other reasons for completing the course included wanting to use the concepts in their own social health initiatives or a pre-existing interest in social health. While all Phase II participants were actively recruited to complete the online CPD by DPG, they were still asked to describe their motivation for completing the CPD. They noted a variety of factors, such as their personal interest in learning more about social determinants of health and the impact social needs can have on patient care. They also wanted to learn more about ways to address health inequities and become more familiar with the resources available to physicians and clinical staff within Kaiser Permanente.

*“Because there's a gap in my knowledge and I want to be a better provider…I have a very high Medicaid population, I have a huge subpopulation of patients who have these …There's a gap there that I know I need to address.” (Phase II – Physician)*

Another motivator for completing the online CPD was the leadership role that some of the physicians maintained. As physician and administrative leads, they considered it a responsibility to review the training content covered, understand how it could be useful for their colleagues within their regions, and translate applicable lessons learned into daily patient care. One physician also noted how the information presented in the course could be incorporated into medical resident training, as well as other relevant trainings.

#### Introduction to new terminology and concepts, case studies based on real-world examples, and role reinforcement are key benefits of the CPD course

Overall, participants reported that the online CPD was easy to follow and provided sufficient time for absorption of the material. For individuals newer to the concepts, the CPD allowed them to glean fundamentals of the social determinants of health, expanding their internal definitions of what contributes to health and healthcare. Providers, who often were more familiar with social determinants of health and its impact on health due to their direct interaction, noted it was still valuable that the online CPD explained the rationale for why social risks are important factors to consider when providing patient care. Physicians stated the CPD’s content and conversation scripting suggestions could be applied daily in patient care and help to facilitate shared decision making among physicians and their patients.

*“I think the module was really helpful in allowing me to think through how to do the shared decision-making, because I am starting to hear those red flags come up in conversation and I'm feeling a little uncomfortable, thinking okay, what next? I hear this information; I hear this clue - I don't really know what to do with this” (Phase II – Physician)*

Participants often mentioning the case studies as a particular strength of the CPD as they felt relatable to their own practice and emphasized the subtle contextual clues that might alert a provider that a patient is experiencing a social risk. Participants also appreciated the diversity represented among the case study characters, which was an intentional decision made in the design and build phases. By showcasing patients and providers from various backgrounds experiencing different social risks and needs, participants felt it addressed their implicit biases around who typically experiences what kinds of social risks.

*“...to show different types of people, different skin colors, [and] age ranges, just demonstrating how social health can impact obviously anyone.” (Phase I – Pharmacist)*

Physicians in particular noted that the case studies included in the training module were very relevant to their daily interactions with their patients. The case studies were also instrumental in demonstrating what a typical physician-patient interaction might look like, and how to ask the right questions in order for the patient to be responsive and understand that resources may be available to help address their needs.

*“I think the case-based nature was nice, just to kind of give examples. I think it was a lot more understandable than just giving principles, [by] having [a] specific…medication and the example of how to modify the treatment plan to address the social needs. So, I think that was really helpful.” (Phase II – Physician)*

Some Phase II physicians noted that another valuable aspect of completing the online CPD was acknowledging the notion that there are multiple ways to adapt care planning to meet the needs of the patient and their social factors and consider the social factors they may be experiencing. They noted that the training content verified the importance of considering a patient’s circumstances when developing a treatment plan and not applying a “one-size-fits-all” approach, aligning with Phase I participants.

*“I would say – it's okay to change what your first-choice treatment plan is, based on the information you receive from a patient about perhaps their challenges and social determinants that we must factor in. It doesn't mean that you're being unethical or not giving best top scope of care. You have to shift sometimes. I think that's something that we widely need to educate providers about, especially I find, very new providers sometimes have the hardest time with that.” (Phase II – Physician)*

Phase I participants also expressed that the CPD reinforced their beliefs that every health professional should be engaged in social health. One important factor identified by physicians was that the training content on social health brought forth the importance of considering a patient’s social factors during a clinical visit, and the value in asking patients questions pertaining to their social circumstances. While participants noted challenges with implementing social risk-informed care due to time constraints, the training served as a good reminder to listen for and recognize the clues that may be associated with patients facing social challenges, and to also apply motivational interviewing skills to solicit this type of information.

*“It's one of these things, that in the back of your mind you know oh yeah, I should be doing that. It's bringing that more into the forefront.”(Phase II – Physician)*

#### A one-size-fits-all approach to training compromises nuance and depth

‘Addressing Social Health in Medicine’ was intentionally designed as a broad training that was relevant in all eight regions across the United States that Kaiser Permanente operates in and for a variety of provider roles. As a result, the most common feedback from participants was a desire for more region- or role-specific training. The specificity would allow CPDs to be more applicable and less abstract, especially for non-provider members of the care team. Additionally, while most participants were satisfied with the case studies, a few felt the case studies were too straightforward. They felt the cases did not reflect enough complexity in medical conditions or social circumstances, such as individuals experiencing homelessness and substance use disorder concurrently.

*“The cases were a little bit more cut and dried, and not necessarily reflecting the messy lives that I witness. I mean when somebody comes in and they're living in a car, it's a totally different vibe than "I just have transportation issues, and oh yeah, mail order pharmacy is going to solve my woes” (Phase I - Physician)*

Physicians also recommended that the CPD should include more examples on how physicians should interact with clinical care team members to address their patients’ social risks, familiarize themselves with the resources available, and learn about different communication styles to elicit this sensitive information from their patients.

*“I think it's always helpful, and I'm not sure I saw as much of this in the module, to give some quick language pointers, a specific way you could ask a question so it's not condescending, not confrontational, normalization – "this is what we ask all our patients, because we want to make sure we're taking good care of you." (Phase II – Physician).*

They noted it would be valuable to have a better understanding of how support services are offered to and accessed by patients within their region for normalizing the concept that social risks impact patients’ ability to care for their health.

*“Maybe the social worker could tell us – I don't know if that's going to be helpful or not, if they tell us ‘These are the referrals we're getting’, ‘this is what we're doing for them’, and if they cannot do it, is there anything else we can do or where are the resources or where we can look for help. Because we want to give patients hope, or at least if we know our resources way better, maybe we can help.” (Phase II – Physician)*

Ultimately, most interviewees supported the use of online CPDs for social health topics in some capacity. However, some cautioned that supplemental interventions are needed to reinforce concepts introduced or covered in online CPDs like ‘Addressing Social Health in Medicine.’ They recognized the importance of proper follow-up to build upon these complex concepts.

*“I think that online modules definitely have a place in all of this, and I see online modules as being most successful and most helpful when it can be combined with other strategies. So, it's not like a one and done, click, I did this.” (Phase I - Physician)*

#### Sustainability of learnings requires an ongoing and systems-level approach

Most Phase I participants indicated additional strategies are necessary to sustain new learnings. Interactive discussion between colleagues was mentioned as meaningful follow up to reinforce new learnings from the module and sustain social risk-informed care. Phase I participants explained that the ability to troubleshoot difficult complex situations together in daily practice would make social risk-informed care more doable in their routine care.

Quick reference guides, such as huddle cards, were recommended by the majority of Phase I participants. Participants felt they would be useful for generating discussion during huddles, reminding team members about important topics and as an easy way to quickly disseminate information especially when huddle cards are already a routine, a known familiar resource by practicing professionals.

Phase I participants also had several recommendations for how this training fits within a broader systems approach to social health integration. In particular, participants felt that access to community health workers and patient navigators would make providers more comfortable asking about social risks. Participants requested more support staff and investment in these resources, including social service resource locators. Most Phase I participants mentioned they used Kaiser Permanente’s internal social service resource locators in some capacity in their region which made clinicians more capable of identifying and addressing social risks and needs.

Phase I providers also specifically mentioned technology can facilitate social risk-informed care and assistance; they provided examples of how they already use electronic health records (EHRs) to streamline social risk-informed care and assistance. They suggested several shortcuts that could be implemented through Health Connect, Kaiser Permanente’s EHR system, and therefore easy to reach during clinic visits. Automated workflows, EHR reminders and Smart Phrases were also suggested and should be explored as practical tools. Leveraging Health Connect to make social risk-informed care and assistance easier for providers was a top priority for physician participants.

Finally, participants expressed that none of the recommended changes can be fulfilled without leadership support. Participants felt that social health initiatives must be prioritized across all regions for any of these recommended changes and improvement in patient care to materialize. From their perspective, leadership should invest more time and resources to allow providers to discuss potentially uncomfortable topics with patients and recover from these emotionally intensive conversations. They suggested that regional and national leadership teams could utilize existing social health work groups to collect feedback on implementation of social health initiatives, which when done iteratively would improve their impact at scale.

*“The more it can become a dialogue of ‘hey, we tried this - what do you think?’ and ongoing small bits of interaction help keep the concepts in peoples’ minds” (Phase I - Physician)*

## Discussion

This evaluation produced several key insights about this online CPD and for the future of social health integration at Kaiser Permanente and other health systems. Firstly, the co-design process resulted in a relevant and high-quality training that resonated with participants. Overall, most participants that completed the post-survey indicated they would recommend the CPD to other professionals, and interview participants praised the CPD for describing the social determinants of health and their importance to overall health, and for providing compelling examples of how social risk-informed care leads to better, more individualized care plans; they also acknowledged that the time allotted for the training facilitated absorption of the material. Providers felt the CPD itself showed realistic examples of what typical interactions look like between patients and providers, and highlighted easy ways they could begin to pay more attention to warning signs that their patients are experiencing a social risk.

Co-design and development in medical education is an important component of person-centered health services and facilitation of shared decision-making [31]. For instance, co-design has been used in other educational materials like the training of mental health professionals to address stigma, [32] to create and test a cross-national midwifery education quality assessment tool, [33] and to generate an eating-and-drinking decision guide for use by family and health professionals caring for hospitalized dementia patients [34]. Shared decision-making is foundational for the success of social risk-informed care and assistance. Our findings reinforce the importance and relevance of this approach for future medical education training development.

The intervention was designed with providers in mind but recognizes that clinical care teams consist of diverse staff, each with unique responsibilities, perspectives, and patient interactions. Interview participants emphasized that their specific roles influence how they engage with patients on social health needs. For future implementation, it is crucial to develop role-specific training modules tailored to each team member’s responsibilities. For example, physicians focus on diagnosing and creating treatment plans, so their training should emphasize how social risks affect medical outcomes and guide them in integrating this information into clinical decisions. Nurses, who often manage ongoing care and patient education, would benefit from training on identifying social risks during patient interactions, building trust, and connecting patients with appropriate resources. Despite the strengths of the CPD, participants highlighted their need for additional support to fully reap the benefits of social risk-informed care and assistance for their patients. Although most providers believe they are already practicing social risk-informed care, prior “secret shopper” audio-recorded visits find that most providers miss opportunities to probe further about social risks patients are facing and adapt care plans to address those risks. [17] Therefore, implementing social risk-informed care involves provider behavior change as it requires physicians and advanced practitioners to adjust the way they interact with and react to patients and families. A prior meta-analysis of CPD literature found that longitudinal, multifaceted and active interventions are more effective at creating and sustaining provider behavior change than single instance, passive or shorter training [35]. These findings were echoed by participants in our study, who felt that a single-instance CPD training will not transform provider behavior across an entire health system, especially when distributed via passive dissemination (I.e. email chains, newsletters, etc.). Passive approaches have long been known to produce lackluster dissemination and implementation results [36, 37]. Alternatively, tailoring implementation and dissemination strategies to overcome context-specific barriers to change and target audience groups has been used successfully in other studies to design and implement clinical decision tools [38].

To move beyond single-instance training towards a more comprehensive approach to social risk-informed care which is more likely to achieve widespread provider behavior change, multi-faceted, longitudinal and targeted interventions are critical [39]. Evaluation participants emphasized the value of sustained, iterative opportunities to both reinforce and build upon the concepts presented in the CPD. Several of the strategies suggested by participants are reflected in the Expert Recommendations for Implementing Change. Strategies such as establishing learning collaboratives, where clinical innovations are introduced in groups across systems of clinicians and are reinforced through shared educational experiences, could be one approach; in Pennsylvania, this strategy was used to successfully introduce and sustain trauma-informed care in clinics across over 20 counties [40]. Mandating change through leadership prioritization is another avenue outlined in ERIC implementation strategies and mentioned by our participants, as well as recruiting local opinion leaders who can organically advocate for the change within the organization and align incentives to help support these changes [41]. Training initiatives have the potential to be more effective when formal and informal leaders demonstrate a commitment to change, as seen in the implementation of a training initiative for person-centered care in community mental health clinics [40].

Our evaluation had several limitations. Firstly, we were unable to link the pre- and post-test survey answers to understand changes in knowledge, attitude, and beliefs about social risk-informed care at the individual provider level. We also were unable to link survey responses to qualitative interview participants. Relative to the interviews, survey respondents were largely non-physicians. Linked records would have given the evaluation team a better understanding of the immediate effectiveness of the training content and more coherence across survey results and interviews. Second, not all interviewees were members of the target audience (providers). While other members of the care team have their own roles in social health integration, interviewing more providers could have elicited additional ideas and themes. Third, our results are subject to selection bias for multiple reasons. In qualitative interviews, Phase II participants were specifically recruited to take the training and provide feedback based on previous engagement in social health work at Kaiser Permanente. Additionally, most participants we interviewed were already interested in social health when they took the training. As a result, our findings are largely within a pool of innovators and early adopters in social health and may not hold with populations less invested in the topic [42]. Fourth, while three co-authors participated in the coding process, co-authors largely independently coded transcripts without overlap. Double-coding of a proportion or all the transcripts would have decreased the risk of individual biases influencing the coding process. Finally, while the evaluation team did reach thematic saturation with our current sample, future studies should consider interviewing larger samples of providers or across multiple healthcare systems to reflect providers with various knowledge, attitudes, and beliefs about social health integration.

While the evaluation had several limitations, this work represents one of the first provider-targeted trainings for social risk-informed care and one of the first evaluations of adjustment-centric CPDs; this work offers lessons for other health systems interested in social health and seeking to apply the NAM’s five priorities for integration of social care. This work also provides an example of using multiple data sources to comprehensively inform a theory and framework-driven evaluation. As health systems and their regulatory bodies increase interest and engagement in social health, as well as screening and assistance programs, training providers to identify and meaningfully utilize social health data to adjust care becomes more critical. For clinicians to effectively use this information to provide social risk-informed care to improve patient outcomes, multi-faceted, targeted, and longitudinal interventions are necessary to support providers in effectively and consistently identifying social risks and adjusting care plans during clinician-patient interactions. Health systems should invest resources specifically into comprehensive training plans for providers to prepare and support them for their role in social health integration.

## Supplementary Information

Additional file 1.

Additional file 2.

## Data Availability

The datasets used and/or analyzed during the current study are available from the corresponding author on reasonable request.
