# Supplementary material for: Lessons learned on social health integration: evaluating a novel social health integration and social risk-informed care online continuing professional development course for primary care providers
Source: BMC Med Educ. 2025 Apr 8;25:496. doi: 10.1186/s12909-025-06971-9 (PMC11977923; doi:10.1186/s12909-025-06971-9)
Supplement: Supplementary file 1 — Additional file 1. [file 12909_2025_6971_MOESM1_ESM.docx]

**Appendix 1.**

Addressing Social Health in Medicine Evaluation Interview Guide

Thank you for completing the online training “Addressing Social Health in Medicine,” and for participating in this follow-up interview! We are looking forward to learning from you about the effectiveness of the training and understanding how these skills can be translated into improving patient outcomes.

I want to remind you that today’s session will be recorded and transcribed but your name and any other personally identifiable information will be removed prior to analysis and reporting.

Our questions today are intended to determine the effectiveness and value of the training module you recently completed as well as understanding more broadly what additional strategies may be necessary to support the adoption and maintenance of social health integration as KP providers.

Before we begin, did you have a chance to review the email entitled “Addressing Social Health In Medicine Refresher”?

*(****IF YES)*** Great! Thank you for taking the time to read the material before our discussion. Did you have any questions about social health integration, or the difference between social risk-informed care and social needs-targeted care?

(answer any questions they have briefly)

(***IF NO***) No problem! As a precursor, I would like to review a few terms to ensure we are discussing the same things throughout the interview. The training module we’re talking about today is part of the social health initiatives at KP. Social health integration is the application of both social risk-informed care and social needs-targeted care into clinical practice.

Social risk-informed care involves adapting or adjusting patients’ treatment plans to accommodate specific social risks they are facing; for example, if a diabetic patient does not have access to stable housing and a reliable refrigerator, the provider and patient could develop a plan of care that incorporates oral medication or insulin pens that do not require refrigeration.

Social needs-targeted care involves activities in clinical settings that seek to address patients’ social risks directly. This includes screening for social needs a patient may have, such as insecure food supply, unstable housing, or unreliable transportation, and connecting them to community organizations or professionals that can provide them with resources to address their specific needs.

(***EVERYONE)***

Your responses will help shape social health dissemination and integration strategies at KP nationally and regionally. If you do not have any further questions, let’s get started!

**Questions**

1. Please briefly describe your role at KP

2. From your perspective, whose role is it to address patients’ social risks or social needs within the care delivery system?

a. What role do you feel you should play, or do you already play, in addressing patient’s social health?

3. Before you took the training, what was your familiarity with social risk-informed care?

a. One example of social risk-informed care is changing a patient’s medication to a cheaper, but still effective, version if they are experiencing financial hardship. Can you think of a similar example from your practice?

b. [if the provider talks about connecting patients to resources (i.e. social needs-targeted care)] what about adapting patient care plans based on social health issues they may be facing?

***IF REPEAT INTERVIEWEE ASK INSTEAD***: Since our interview last year, do you feel like you have come across more or taken part in any social health initiatives, and in particular social risk-informed care, within your region or at the national level?

**a.** If yes, can you provide some examples?

b. (if the provider says they haven’t seen more about social health initiatives) why do you think that is?

4. How did you learn about the training?

a. I’m going to drop a list of resources into the chat. Which of these do you use for accessing more information about social health either within or outside of KP? Are there any other resources you use besides the ones listed?

***DROP IN THE CHAT THE FOLLOWING:*** listservs, websites, workgroups, colleagues, specific subject matter experts, etc *(Note: national is particularly interested in what communication channels can be used to disseminate resources/information)*

*b.* Would you recommend using online training modules such as this one to learn new information about social health topics?

5. What motivated you to pursue the training?

a. How easy or difficult was it to access the training through KP Learn?

b. Besides CME credits, what approaches do you think would[BB10] work best to recruit providers to take the module?

6. What were your key takeaways from the training?

a. What did you like about it?

***IF KPWA ASK INSTEAD*** : Given that you have already been involved in social health integration in your clinical setting, did you find the training useful? Does it fit with your current experience and add value to your practice?

1. (if yes) how so?

2. (if no, probe further as to why)

3. (Optional probe) From your perspective, what else is needed for social risk informed care to be an effective and sustainable part of social health integration in your region?

7. What would you change or improve about the training?

a. what about the training wasn’t useful or helpful?

8. After completing the training, has your opinion or perspective changed on the importance of addressing your patients’ social health in your clinical practice?

(if yes) how so?

(if no, probe further as to why)

9. Have you changed anything about how you approach patient care since completing the training?

(if yes) how so? Can you provide an example?

a. (if no, probe further as to why)

(*If the provider discusses barriers in clinical infrastructure and community)* what else could be in the way?

10. What kind of support do you and your colleagues need to consistently practice social risk-informed care?

*a.* What type of support is needed from leadership at the regional and national levels to make this a priority and part of standard of care?

*b.* What would make it easier to implement and sustain social risk-informed care and why?

***IF REPEAT INTERVIEWEE HAS PRACTICED SRIC SINCE LAST INTERVIEWED, ASK INSTEAD:*** Did your practice of social risk-informed care change over the past year? If yes, how so? Why? What made it easier or harder to consistently incorporate social risk-informed care into your practice?

11. I’m going to drop a list of follow-up methods into the chat. Which of these would be the most valuable for providers in your department to reinforce the practice of social risk-informed care? Is there something else that would be useful that is not on this list?

(***DROP IN THE CHAT THE FOLLOWING:*** *Overview of community resources and/or Thrive Local, huddle cards, additional training at certain intervals, role play scenarios, review case studies, etc)*

12. Is there anything else you would like to add before we conclude our time together?

Thank you so much for taking the time today to answer our questions. Your answers will guide the implementation and dissemination of social health initiatives at KP both regionally and nationally. If you have any questions at a later time, please do not hesitate to reach out.
