# Supplementary material for: Lessons learned on social health integration: evaluating a novel social health integration and social risk-informed care online continuing professional development course for primary care providers
Source: BMC Med Educ. 2025 Apr 8;25:496. doi: 10.1186/s12909-025-06971-9 (PMC11977923; doi:10.1186/s12909-025-06971-9)
Supplement: Supplementary file 2 — Additional file 2. [file 12909_2025_6971_MOESM2_ESM.docx]

**Appendix 2: SRIC Interview Guide Codebook**

The parent codes, child codes and grandchild codes are structured so that each child code should also be labeled as its parent code of origin, and similarly for grandchild codes.

For example, if a provider says “I don’t have enough time with patients to practice medicine like in the module” it would be coded as *Skills_barriers_Time, Skills_Barriers, & Skills –* in that way we can easily select all excerpts labeled as barriers at once, or facilitators and barriers related to the Parent code *Skills.*

| **Code Name** | **Code Type** | **Description** |
| --- | --- | --- |
| **SRIC_Role** | **Parent** | the professional role the provider believes should be primarily responsible for addressing patients’ social risks (may also include reasons) |
| SRIC_Role_Provider | Child | The provider believes providers should be primarily responsible for addressing patients’ social risks in medicine (may also include reasons) |
| SRIC_Role_Social | Child | The provider believes social service roles, such as social work or case management, should be primarily responsible for addressing patients’ social risks in medicine (may also include reasons) |
| SRIC_Role_Other | Child | The provider believes another profession should be primarily responsible for addressing patients’ social risks in medicine (i.e. nurses, community health workers, etc) (may also include reasons) |
| **SNTC_Success** | **Parent** | The provider successfully connects patients to community resources (I.e. home health, food or transport subsidization, community case management, etc) AND the patients received the intended benefit from the connection |
| **SNTC_Failure** | **Parent** | The provider unsuccessfully attempts to connect patients to community resources OR once the patient is connected they do not receive the expected benefits |
| **Dissemination_Channel** | **Parent** | The provider identifies the kinds of resources that they use to find new opportunities or trainings, including the SRIC module |
| Dissemination_Listserv | Child | The provider uses listservs, and/or found the SRIC module through a listserv |
| Dissemination_Workgroups | Child | The provider uses workgroups, and/or found the SRIC module through a workgroup |
| Dissemination_Colleagues | Child | The provider gets information from colleagues, and/or found the SRIC module through a colleague |
| Dissemination_Leader | Child | The provider gets information from a clinic leader, KP or a social health integration leader, and/or found the SRIC module through them |
| **CME_SRIC** | **Parent** | The provider mentions CME credit as a motivating factor in completing the SRIC module |
| **Education** | **Parent** | The provider mentions their access and use of educational materials, such the SRIC module |
| Education_Online | Child | The provider mentions routinely using online trainings, such as the SRIC module, as a way to receive continuing education |
| Education_KPLearn | Child | The provider mentions routinely using KP Learn as a way to receive continuing education |
| Education_Lunch_n_Learn | Child | The provider mentions routinely attending lunch n’ learn sessions as a way to receive continuing education |
| Education_Other | Child | The provider mentions other methods they use to routinely receive continuing education |
| **Module_Changes** | **Parent** | Provider’s recommendations for changes to the SRIC module |
| **Module_Positives** | **Parent** | what the provider likes about the training |
| **Module_Negatives** | **Parent** | What the provider dislikes about the training |
| **Knowledge** | **Parent** | The provider shares new knowledge they gained after taking the SRIC module |
| **Confidence_improved** | **Parent** | The provider mentions their confidence in practicing SRIC improving as a result of the module |
| **Confidence_low** | **Parent** | The provider is not confident in their ability to translate the SRIC skills in their practice |
| **Attitude_Positive** | **Parent** | The provider’s attitude changed positively toward social health integration, or SRIC specifically, due to the module |
| **Attitude_Neutral** | **Parent** | The provider’s attitude didn’t change toward social health integration, or SRIC specifically, after taking to the module |
| **Skills** | **Parent** | The provider mentions translating the SRIC skills to their clinical practice |
| Skills_Barriers | Child | Barriers to the provider adopting and practicing SRIC skills into their clinical practice |
| *Skills_Barriers_Time* | *Grandchild* | Time is identified as a barrier to adopting SRIC (with patients, to chart, in general, etc) |
| *Skills_Barriers_Infrastructure* | *Grandchild* | Clinical infrastructure is identified as a barrier to adopting practicing SRIC |
| *Skill_Barriers_Culture* | *Grandchild* | Clinic or organizational culture is identified as a barrier to adopting and practicing SRIC |
| *Skills_Barriers_COVID* | *Grandchild* | The pandemic, or necessary precautions taken in response to the pandemic, is identified as a barrier to adopting and practicing SRIC |
| *Skills_Barriers_Technology* | *Grandchild* | Provider mentions technology, such as telehealth or EHRs, as barriers to adopting and practicing SRIC |
| *Skills_Barriers_Other* | *Grandchild* | Other things are identified as barriers to adopting and practicing SRIC |
| Skills_Facilitators | Child | Barriers to the provider adopting SRIC skills in their clinical practice |
| *Skills_Facilitators_Champions* | *Grandchild* | SRIC champions are identified as facilitators to adopting and practicing SRIC |
| *Skills_Facilitators_Technology* | *Grandchild* | Technology, such as telehealth or EHRs, is identified as a facilitator to adopting and practicing SRIC |
| *Skills_Facilitators_Other* | *Grandchild* | Other things are identified as facilitators to adopting and practicing SRIC |
| Skills_Example | Child | The provider gives an example of accurately and effectively practicing SRIC after completing the module |
| Skills_Prior | Child | The provider mentions, and/or provides examples of, skills they used to address social risks in medicine prior to taking the module |
| Skills_Sustainability | Child | The provider mentions the ability (or lackthereof) to consistently and effectively address social health in medicine over time |
| **Additional_Resources** | **Parent** | Additional resources desired to support providers’ adoption and practice of SRIC |
| Additional_Resources_Community | Child | The provider identifies an overview or list of community resources as a useful additional resource in practicing SRIC |
| Additional_Resources_ThriveLocal | Child | The provider identifies Thrive Local as a useful additional resource in practicing SRIC |
| Additional Resources_other | Child | The provider identifies other resources that would be useful for practicing SRIC |
| **Other_Initiatives** | **Parent** | The provider mentions how the module integrates with other social health initiatives, including social needs screening |
| **FollowUp** | **Parent** | Follow up resources that would support providers in consistently practicing SRIC |
| FollowUp_training | Child | Additional trainings, either online or in person, are identified as either useful or not useful follow up to support consistent practice of SRIC |
| FollowUp_roleplay | Child | Additional role-play scenarios are identified as either useful or not useful follow up to support consistent practice of SRIC |
| FollowUp_practice_facilitation | Child | Practice facilitation (I.e. weekly meetings with consultant) is identified as either useful or not useful follow up to support consistent practice of SRIC |
| FollowUp_collaborative | Child | Peer quality improvement collaboratives are identified as either useful or not useful follow ups to support consistent practice of SRIC |
| FollowUp_visits | Child | Site visits to high performing clinics are identified as either useful or not useful follow ups to support consistent practice of SRIC |
